# Tumor-Associated Neutrophils as a New Prognostic Factor in Cancer: A Systematic Review and Meta-Analysis

**DOI:** 10.1371/journal.pone.0098259

**Published:** 2014-06-06

**Authors:** Meixiao Shen, Pingping Hu, Frede Donskov, Guanghui Wang, Qi Liu, Jiajun Du

**Affiliations:** 1 Institute of Oncology, Provincial Hospital Affiliated to Shandong University, Shandong University, Jinan, P. R. China; 2 Department of Oncology, Aarhus University Hospital, Aarhus, Denmark; 3 Department of Thoracic Surgery, Provincial Hospital Affiliated to Shandong University, Shandong University, Jinan, P. R. China; University of North Carolina School of Medicine, United States of America

## Abstract

**Purpose:**

Tumor-associated neutrophils (TAN) have been reported in a variety of malignancies. We conducted an up-to-date meta-analysis to evaluate the prognostic role of TAN in cancer.

**Method:**

Pubmed, Embase and web of science databases were searched for studies published up to April 2013. Pooled hazard ratios (HRs) and their corresponding 95% confidence intervals (CIs) were calculated. The impact of neutrophils localization and primary antibody were also assessed.

**Results:**

A total of 3946 patients with various solid tumors from 20 studies were included. High density of intratumoral neutrophils were independently associated with unfavorable survival; the pooled HRs were 1.68 (95%CI: 1.36–2.07, I^2^ = 55.8%, *p*<0.001) for recurrence-free survival (RFS)/disease-free survival (DFS), 3.36 (95%CI: 2.08–5.42, I^2^ = 0%, *p*<0.001) for cancer-specific survival (CSS) and 1.66 (95%CI: 1.37–2.01, I^2^ = 70.5%, *p*<0.001) for overall survival (OS). Peritumoral and stromal neutrophils were not statistically significantly associated with survival. When grouped by primary antibody, the pooled HRs were 1.80 (95%CI: 1.47–2.22, I^2^ = 67.7%, *p*<0.001) for CD66b, and 1.44 (95%CI: 0.90–2.30, I^2^ = 45.9%, *p* = 0.125) for CD15, suggesting that CD66b positive TAN might have a better prognostic value than CD15.

**Conclusion:**

High levels of intratumoral neutrophils are associated with unfavorable recurrence-free, cancer-specific and overall survival.

## Introduction

In addition to cancer cells, a tumor lesion contains a number of recruited cells that contribute to the hallmarks of cancer by creating the tumor microenvironment [1]. Stromal cells, blood vessels and infiltrating inflammatory cells are major components of the tumor microenvironment [2]. These cells enable and sustain most of the hallmarks of cancer through reciprocal communications with neoplastic cancer cells [3]. A leukocyte infiltrate, comprising mast cells, T cells, natural killer cells (NK), T-regulatory cells (T-regs), myeloid derived suppressor cells (MDSC), tumor-associated macrophages (TAM) and tumor-associated neutrophils (TAN) are the key participants of the tumor microenvironment where they can promote or inhibit cancer formation and development [4] [5].

Neutrophil granulocytes are the most abundant circulating leukocytes and represent the first line of immune defense against invading pathogens. The role and characteristics of TAN in cancer are poorly defined and has been considered negligible until recently because of their short life span and fully differentiated phenotype [4]. The first report of peripheral blood neutrophils associated with short 5-year survival in humans was published in 1970 [6]. The first study to identify the presence of TAN by use of immunohistochemistry (IHC) as an independent poor prognostic factor in humans and to incorporate TAN into a prognostic risk model based on established clinicopathological features was published in 2006 [7].

Recently, the prognostic role of TAN has been associated with poor clinical outcome in several human cancers, most notably in renal cell carcinoma (RCC) [7], [8], melanoma [9], colorectal cancer (CRC) [10], hepatocellular carcinoma (HCC) [11]–[14], intrahepatic cholangiocarcinoma (ICC) [15], gastric [16], pancreatic ductal carcinoma (PDC) [17] and head and neck cancer (HNC) [18]. However, other studies have demonstrated no relationships between TAN and unfavorable prognosis [19]–[22]. Although compelling, these finding are limited by retrospective design or a single tumor type. In fact, the overall level-1 evidence supporting an excess mortality in patients with high levels of TAN is lacking. Therefore, we performed the first meta-analysis to evaluate the effect of TAN in patients with cancer.

## Materials and Methods

### Search identification

PubMed, Embase and Web of Science were used to search for the original articles analyzing the prognostic value of TAN in human cancer, by means of keywords variably combined: ‘neutrophil’, ‘tumor-infiltrating neutrophils’, ‘tumor-associated neutrophils’, ‘polymorphonuclear neutrophils’, ‘cancer’, ‘carcinoma’, ‘tumor’, ‘prognosis’, ‘prognostic’, and ‘clinical outcome’. Last search was updated on 25 April 2013, and no lower date limit was used. We also searched for references from the bibliographies of all eligible studies and relevant systematic reviews. At the same time, we contacted the authors of eligible articles whenever the essential data were unavailable from original literatures.

### Eligibility criteria

Inclusion and exclusion criteria were made before any meta-analysis of the data. Studies had to meet the following criteria: (1) trials had to deal with human cancer; (2) measure the expression of neutrophils in the tumor tissue; (3) evaluate the association between TAN and the outcome of patients with sufficient details to permit calculation of the hazard ratios (HRs) of each outcome and their 95% confidence intervals (CIs); (4) TAN was dichotomized as ‘high’ and ‘low’ value or equivalent cut-off value; (5) published as a full paper in English. When part or all of the patients were involved in more than one publication, only the most complete or most informative study was included in this analysis.

### Data extraction

All the potentially relevant papers were reviewed and extracted independently by two investigators (MX. S. and PP. H.), and the disagreements were resolved by discussion and the controversial parts were adjudicated by a third investigator (JJ. D). In order to ensure the quality of the meta-analysis, we followed the guidelines provided by the Preferred Reporting Items for Systematic Reviews and Meta-Analyses (PRISMA) statement [23]. Information retrieved from the researches included author, publication year, country of population, sample size, histology, stage, primary antibody, neutrophils location, cut-off criteria, cut-off value, follow-up time, HR and survival analysis.

### Statistical analysis

For the quantitative aggregation of the survival results, we measured the prognostic value of TAN by the HRs and the associated 95% CIs. An extracted HR>1 indicated worse outcome for high level of TAN. The result was considered statistically significant only when the 95% CIs did not overlap with 1. In assessed trials, if HRs and 95% CIs were neither reported directly, nor provided by the authors after request, they were estimated where possible according to the Tierney’s methods [24] (N = 7).

We used the I^2^ statistics to detect the heterogeneity across different subgroups. Pooled estimates of the prognostic value of TAN were calculated by using the random effect model depending on obvious heterogeneity with I^2^>50%; otherwise, the fixed effect model was used. Assessment of publication bias was performed using a funnel plot with Begg’s test and the trim-and-fill analysis. A sensitivity analysis was also conducted to test the impact of the outcomes from these eligible studies. All statistical tests were two-sided and differences at *p*<0.05 were considered statistically significant. Stata Statistical Software (version 12.0 Stata Corp., College Station, TX, USA) was used for all analyses in our analysis.

## Results

### Identification of relevant studies

**Figure 1:**
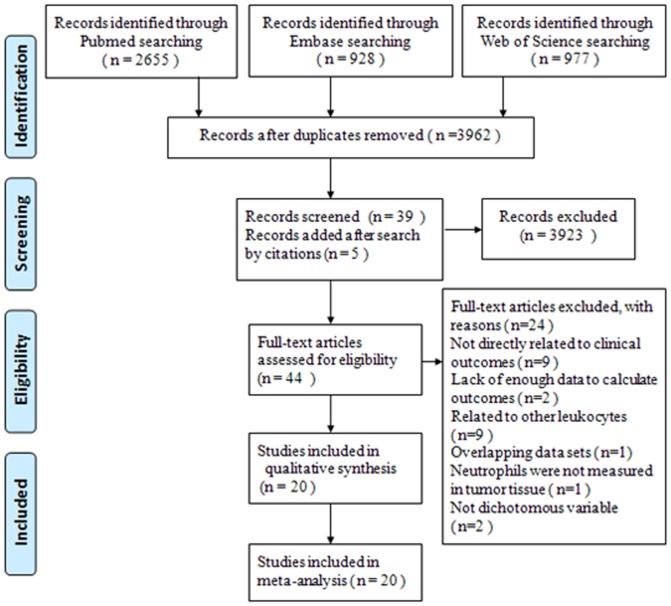
Follow diagram of the meta-analysis.

Totally 3962 potentially relevant studies were identified after initial searches. Based on titles and abstracts screening 3923 studies were excluded, and the remaining 39 were evaluated in full text. After reviewing the bibliographies of these 39 studies, we added 5 studies to the meta-analysis. Of the 44 candidate studies, 9 studies were not directly related to specific outcomes; 2 studies did not provide enough data to calculate the survival data; 9 studies were related to tumor-infiltrating macrophages, eosinophils or lymphocytes instead of neutrophils; only 1 study reported overlapping data; 2 studies did not evaluate the expression of TAN as dichotomous variables; 1 study regarding bronchioloalveolar carcinoma was excluded because neutrophils were measured in bronchoalveolar lavage fluid rather than tumor tissue. Consequently, 20 articles with a total number of 3946 patients were accepted for our meta-analysis (Fig. 1).

### Characteristics of included studies

The characteristics of retained studies are listed in Table 1. In our analysis, neutrophils were further classified into three groups according to the localizations of neutrophils within the tumor compartments: intratumoral (in tumor nests), peritumoral (an area of stroma adjacented to tumor nests was denoted as peritumoral, if at least one tumor cell was observed in the field of view), and stromal (an area was denoted as stromal if no tumor cells were observed in the peritumoral stroma tissue field of view) [25]. Sample size referred to the number of patients assessed for TAN.

**Table 1:** Characteristics of included studies.

First Author	Year	Country of population	Sample size	Histology	Stage	First- antibody	Neutrophils location	Cut-off criteria	Cut-off value	Follow-up time	Hazard ratios	Survival analysis
Rosario Alberto Caruso	2002	Italy	273	Gastric	Ib- IV	- (HE)	Intratumoral	Mean	>10cells/20HPF	NM	Report^a^	OS
Frede Donskov	2006	Denmark	85	RCC	NM	CD66b	Intratumoral	Median	0 cells/mm^2^	Median:57m(32–73 m)	Report^a^, Author^a^	OS
Hanne Krogh Jensen	2009	Denmark	121	RCC	I-IV	CD66b	Intratumoral	Median	0 cells/HPF	Median:124 m (74–194 m)	Report^a+b+c^, Author^a^	RFS, OS, CSS
Sokratis Trellakis	2010	Germany	40	HNC	III -IV	CD66b	Intratumoral	NM	Low: −,+; High: ++,+++	Median:69 m (43–124m)	SR ^a^	OS
Dong-Ming Kuang	2010	China	200	HCC	I-III	CD15	Intratumoral, Peritumoral, Stromal	Median	Intratumoral:4; Peritumoral:54; Stromal:10	NM	SR^a+d^, Survival curve^a+d^	DFS, OS
Yi-Wei Li	2011	China	281	HCC	I-III	CD66b	Intratumoral Peritumoral	Fixed	Intratumoral:70%, Peritumoral:50%	Median:29 m (1.5–83m)	Report^a+c^, Survival curve^a+c^	RFS, OS
Lih-Chyang Chen	2012	China	140	HNC	NM	- (HE)	Intratumoral	Mean	10 neutrophils/ 100 epithelial cells	NM	Survival curve^c^	RFS
Qiang Gao	2012	China	240	HCC	NM	CD66b	Intratumoral	Median	12 cells/ mm^2^	NM	Report^c^	RFS
Fang-Ming Gu	2012	China	123	ICC	I/II/IIIa/ IIIc	CD66b	Intratumoral, Peritumoral	Median	86 cells/ mm^2^	Median:13m (4–111m)	Report^a^	OS
Marius Ilie	2011	France	632	NSCLC	I – III	CD66b	Intratumoral	Median	49 cells/mm^2^	Median:30m (0–112 m)	Survival curve^a^	OS
Trine O. Jensen	2011	Denmark	186	Melanoma	I/II	CD66b	Intratumoral	Fixed	0 cells/2 HPF	Median:12.2y (10.4–14.2 y)	Report^a+b+c^	RFS,OS, CSS
Hui-Lan Rao	2012	China	229	CRC	I-IV	CD66b	Intratumoral	Mean	60 per TMA spot	Average:55.4m Median:60.0m (0.5–98 m)	Report^a^, Survival curve^a^	OS
CH Richards	2012	UK	130	CRC	NM	- (HE)	Peritumoral	Median	High: score 2–3, Low: score 0–1	Median:105m (55–163m)	DE^b^	CSS
Jing-jing Zhao	2012	China	115	Gastric	I-IV	CD15	Intratumoral	Median	21.60 cells/HPF	NM	Report^a^	OS
Shao-Lai Zhou	2012	China	323	HCC	NM	CD66b	Intratumoral	NM	Hgih, Low	NM	Report^a+c^	OS, RFS
Andreas Carus	2013	Denmark	101	CC	IB-IIA	CD66b	Intratumoral, Peritumoral, Stromal	Median	Intratumoral:23.2cells/mm^2^, Peritumoral:53.1cells/mm^2^, Stromal:28.3cells/ mm^ 2^	NM	Author^c^, Report^c^	RFS
Andreas Carus	2013	Denmark	335	NSCLC	I–IIIA	CD66b	Intratumoral, Peritumoral, Stromal	Median	Intratumoral:8.7cells/mm^2^, Stromal:21.0 cells/ mm^2^	NM	Author^a+c^	OS, RFS
Claudia A. Dumitru	2013	Germany	97	HNC	NM	CD66b	Intratumoral	Median	NM	NM	Survival curve^a^	OS
Claudia A. Dumitru	2013	Germany	83	HNC	I-IV	CD66b	Intratumoral	NM	High: medium and strong, Low: negative, weak	NM	Report^a^	OS
Y Ino	2013	Japan	212	PDC	NM	CD66b	Intratumoral	Median	NM	Median:18.8m (2.6–201m)	Report^a+d^	OS, DFS

Abbreviations: Gastric: gastric carcinoma; RCC: renal cell carcinoma; HNC: head and neck carcinoma; HCC: hepatocellular carcinoma; ICC: intrahepatic cholangiocarcinoma; NSCLC: non-small-cell lung cancer; CRC: colorectal carcinomas; CC: cervical cancer; PDC: pancreatic ductal carcinoma; HE: hematoxylin-eosin staining; OS: overall survival; CSS: cancer-specific survival; RFS: recurrence-free survival; DFS: disease-free survival; NM: not mentioned; a: OS; b: CSS; c: RFS; d: DFS; m: months; y: years; DE: data extrapolated; SR: systematic review [36].

Eligible studies included 5 studies for HCC (n = 1044) and ICC (n = 123) [11]–[15], 4 studies for HNC (n = 360) [18], [21], [22], [26], 2 studies for non-small-cell lung cancer (NSCLC, n = 967) [20], [27], 2 studies for RCC (n = 206) [7], [8], 2 studies for gastric carcinoma (n = 388) [16], [19], 2 studies for CRC (n = 359) [10], [28] and 1 study for cervical carcinoma (CC, n = 101) [29], melanoma (n = 186) [9] and PDC (n = 212) [17], respectively.

Clinical outcomes were assessed in each study. 16 studies including 3353 patients had overall survival (OS) as the primary endpoint [7]–[12], [14]–[22], [27]. All of 16 studies assessed intratumoral neutrophils; 2 studies assessed peritumoral neutrophils; and 2 studies assessed stromal neutrophils. One study assessed various neutrophil localizations in tumor compartment and had various survival endpoints was included in the analyses repeatedly [30].

10 studies including 2139 patients reported data on recurrence-free survival (RFS)/disease-free survival (DFS) [8], [9], [11]–[14], [17], [20], [26], [29]. All of the 10 studies assessed intratumoral neutrophils, 3 studies assessed peritumoral neutrophils, and 3 studies assessed stromal neutrophils. In addition, 3 studies [8], [9], [28] including 437 patients had cancer-specific survival (CSS) as an endpoint.

Regarding the method to identify TAN, 17 studies used IHC to detect the expression of TAN. The monoclonal antibody against CD66b was used in 15 studies, and the monoclonal antibody against CD15 was used in 2 studies. Hematoxylin-eosin (H&E) stain was used in 3 studies. Neutrophils were assessed by using stereology (5 studies), automatic computerized quantification (4 studies) and semiquantitative methods (11 studies). The expression of TAN was dichotomized into high and low levels or present and absent groups, and the cut-off values for 20 trials were listed in Table 1. A total of 15 studies used the median or mean levels as cut-off values, 2 studies used fixed cut-off values and 3 studies did not mention the detailed information about cut-off criteria.

### Meta-analysis

Among the 20 studies, 9 studies presented HRs and 95% CIs for both unadjusted and adjusted classifiers. When both unadjusted and adjusted HRs were extracted from the original papers, the adjusted HRs were included into our analysis. This analysis was denoted as the maximally adjusted association analysis [31], which we mainly discussed below. Inversely if the unadjusted HRs were included into meta-analysis, this analysis was denoted as crude (unadjusted) analysis [31]. All calculated pooled HRs were shown in Table 2. However, there was no obvious difference in the pooled HRs between the maximally adjusted association analysis and the crude analysis.

**Table 2:** All calculated pooled HRs in meta-analysis.

GROUP	AREA	OS		RFS/DFS		CSS	
		HR(95%CI)	I^2^	HR(95%CI)	I^2^	HR(95%CI)	I^2^
All cancers^a^	Intratumoral	1.66(1.37–2.01)	I^2^ = 70.5%	1.68(1.36–2.07)	I^2^ = 55.8%	3.36(2.08–5.42)	I^2^ = 0%
	Peritumoral	1.66(0.64–4.32)	I^2^ = 87.2%	1.80(0.96–3.37)	I^2^ = 73.5%	ND	ND
	Stromal	1.10(0.76–1.61)	I^2^ = 56.8%	1.27(0.75–2.16)	I^2^ = 76.8%	ND	ND
HCC and ICC^a^	Intratumoral	1.80(1.33–2.43)	I^2^ = 57.7%	1.58(1.33–1.88 )	I^2^ = 36.9%	ND	ND
HNC^a^	Intratumoral	1.69(1.10–2.60)	I^2^ = 0%	ND	ND	ND	ND
NSCLC^a^	Intratumoral	1.16(1.00–1.35)	I^2^ = 0%	ND	ND	ND	ND
RCC^a^	Intratumoral	2.69(1.89–3.83)	I^2^ = 0.0%	ND	ND	ND	ND
Gastric^a^	Intratumoral	1.20(0.50–2.89)	I^2^ = 84.6%	ND	ND	ND	ND
CD66b^a^	Intratumoral	1.80(1.47–2.22)	I^2^ = 67.7%	ND	ND	ND	ND
CD15^a^	Intratumoral	1.44(0.90–2.30)	I^2^ = 45.9%	ND	ND	ND	ND
All cancers ^b^	Intratumoral	1.85(1.42–2.40)	I^2^ = 85.1%	1.68(1.20–2.34)	I^2^ = 80.6%	4.99(3.13–7.95)	I^2^ = 10.1%
	Peritumoral	1.28(0.62,2.66)	I^2^ = 87.8%	1.78(0.97–3.28)	I^2^ = 81.8%	ND	ND
	Stromal	1.10(0.76,1.61)	I^2^ = 56.8%	1.27(0.75–2.16)	I^2^ = 76.8%	ND	ND

Abbreviations: HCC: hepatocellular carcinoma; ICC: intrahepatic cholangiocarcinoma; HNC: head and neck carcinoma, NSCLC: non-small-cell lung cancer; RCC: renal cell carcinoma; Gastric: gastric carcinoma; ND: not done. OS: overall survival; CSS: cancer-specific survival; RFS: recurrence-free survival; DFS: disease-free survival; a: maximally adjusted association HR ( When both unadjusted and adjusted HRs were extracted from the original papers, the adjusted HRs were included into analysis ); b: crude association HR ( When both unadjusted and adjusted HRs were extracted from the original papers, the unadjusted HRs were included into our analysis).

**Figure 2:**
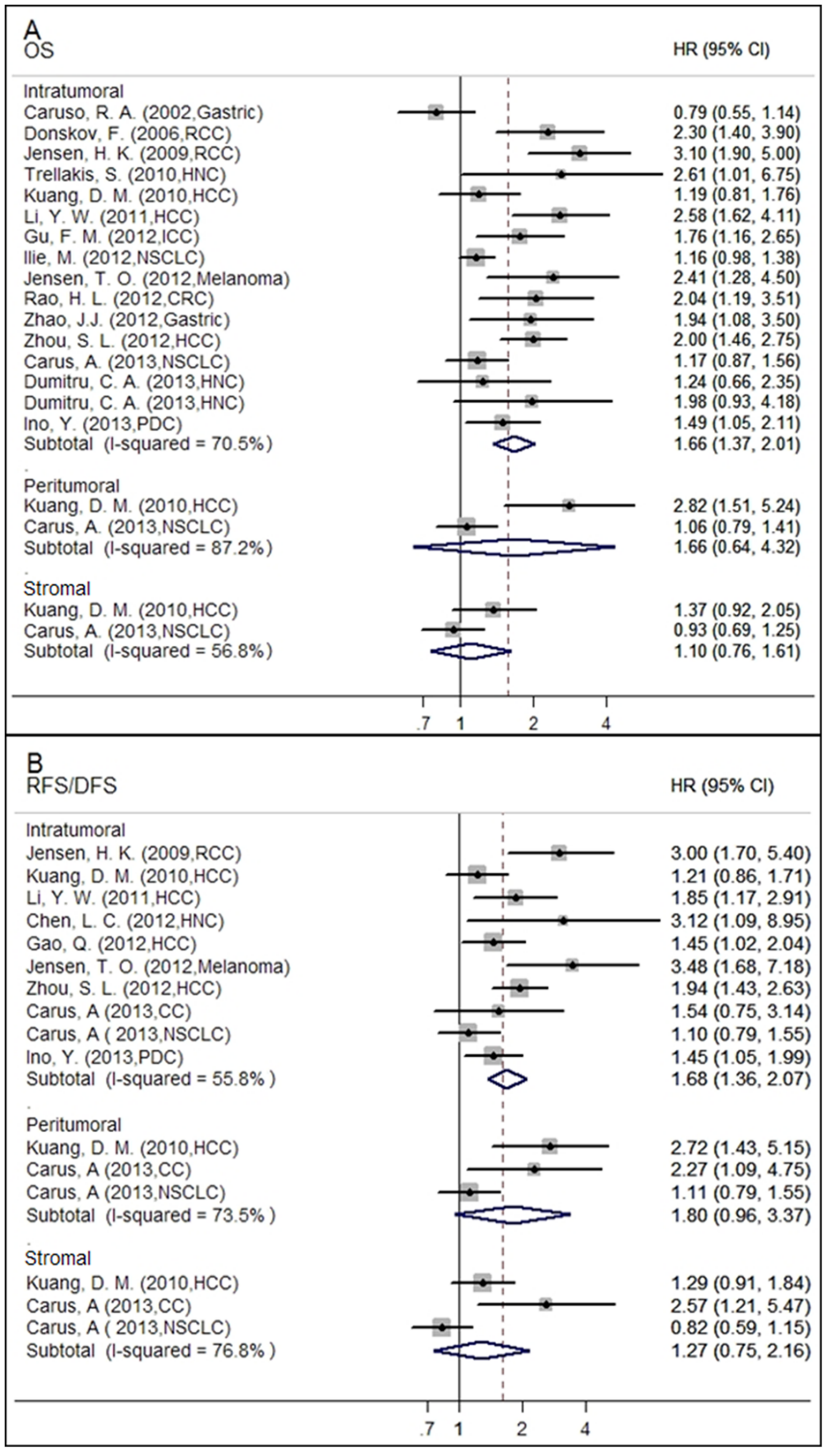
Forrest plots evaluating maximally adjusted association between TAN and clinical outcomes in all cancers. (A) Forrest plot to assess the overall effect of TAN on OS in all cancer patients. (B) Forrest plot to assess the overall effect of TAN on RFS/DFS in all cancer patients.

Neutrophils were divided into intratumoral, peritumoral, and stromal in all studies, with survival analyses evaluating OS, CSS, RFS and DFS. For quantitative analyses, we calculated the pooled HRs for all available trials grouped by OS, RFS/DFS, and CSS. Meta-analysis assessing OS as the endpoint were performed in 16 studies, the pooled HR of intratumoral neutrophils was 1.66 (95%CI: 1.37–2.01, I^2^ = 70.5%) (Fig. 2 A), indicating that high densities of intratumoral neutrophils were independently associated with short survival in various cancers. High densities of neutrophils presented in other areas of the tumor compartments had no statistically significantly association with OS; the combined HRs were 1.66 (95%CI: 0.64–4.32, I^2^ = 87.2%) for peritumoral and 1.10 (95%CI: 0.76–1.61, I^2^ = 56.8%) for stromal neutrophils respectively (Table 2).

High intratumoral neutrophil levels were significantly associated with short RFS/DFS (HR = 1.68, 95%CI: 1.36–2.07, I^2^ = 55.8%), whereas peritumoral (HR = 1.80, 95%CI: 0.96–3.37, I^2^ = 73.5%), and stromal (HR = 1.27, 95%CI: 0.75–2.16, I^2^ = 76.8%) were not (Fig. 2B). Elevated intratumoral neutrophils densities were also significantly associated with poor CSS (HR = 3.36, 95%CI: 2.08–5.42, I^2^ = 0%) (Table 2).

**Figure 3:**
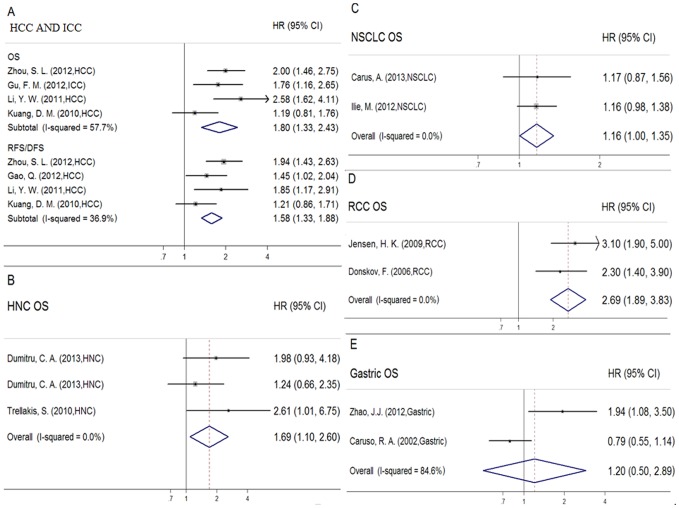
Forrest plots evaluating maximally adjusted association between intratumoral neutrophils and clinical outcomes in subgroups. HRs of HCC patients are reported as (A). HRs of HNC patients are reported as (B). HRs of NSCLC patients are reported as (C). HRs of RCC patients are reported as (D). HRs of Gastric carcinoma patients are reported as (E).

Subsequently, in order to further investigate the prognostic role of intratumoral neutrophils in different types of cancers, we grouped eligible studies by cancer types. In HCC and ICC, data demonstrated high intratumoral neutrophils as a prognostic indicator of unfavourable prognosis compared to those with low levels, the pooled HR = 1.80 for OS (95%CI: 1.33–2.43, I^2^ = 57.7%), and HR = 1.58 (95%CI: 1.33–1.88, I^2^ = 36.9%) for RFS/DFS (Fig. 3A). High density of TAN was associated with short OS in HNC (HR = 1.69, 95%CI: 1.10–2.60, I^2^ = 0%) (Fig. 3B), in NSCLC (HR = 1.16, 95%CI: 1.00–1.35, I^2^ = 0%) (Fig. 3C), in RCC (HR =  2.69, 95%CI: 1.89–3.83, I^2^ = 0%) (Fig. 3D), but not in gastric carcinoma (HR =  1.20, 95%CI: 0.50–2.89, I^2^ = 84.6%) (Fig. 3E). Owing to the limitation of the number of eligible studies, the subgroup analyses of melanoma, CC, PDC, and CRC were not performed.

To evaluate the effect of the primary antibody, studies were grouped according to the primary antibody used. The pooled HR of the studies evaluating OS for intratumoral neutrophils was 1.80 (95%CI: 1.47–2.22, I^2^ = 67.7%) for CD66b, and 1.44 (95%CI: 0.90–2.30, I^2^ = 45.9%) for CD15 (Table 2).

Heterogeneity was observed among the 16 indepedent trials which had OS as the endpoint (I^2^ = 70.5% for intratumral, I^2^ = 87.2% for peritmoral, and I^2^ = 56.8% for stromal neutrophils), as well as 10 independent trials which had RFS/DFS as the endpoint (I^2^ = 55.8% for intratumral, I^2^ = 73.5% for peritmoral, and I^2^ = 76.8% for stromal neutrophils). In subgroup analyses of tumor types, heterogeneity was seen in HCC and ICC (I^2^ = 57.7%) and gastric carcinoma (I^2^ = 84.6%) evaluating OS for intratumoral neutrophils while no heterogeneity was observed in other subgroups.

### Publication bias and Sensitivity analysis

Publication bias was assessed by a funnel plot with Begg’s test and the trim-and-fill analysis. Among involved 16 studies evaluating OS for intratumoral neutrophils, the *p* value was 0.260 for Begg’s test. And the trim-and-fill analysis imputed 5 studies, which would not alter the results. These methods suggested that no publication bias were observed in the subgroup evaluating OS for intratumoral neutrophils. Moreover, our data indicated that no publication bias were observed among 10 studies evaluating RFS/DFS and intratumoral neutrophils (*p* = 0.152 for Begg’s test; the trim-and-fill analysis imputed 2 studies, which would not alter the results).

**Figure 4:**
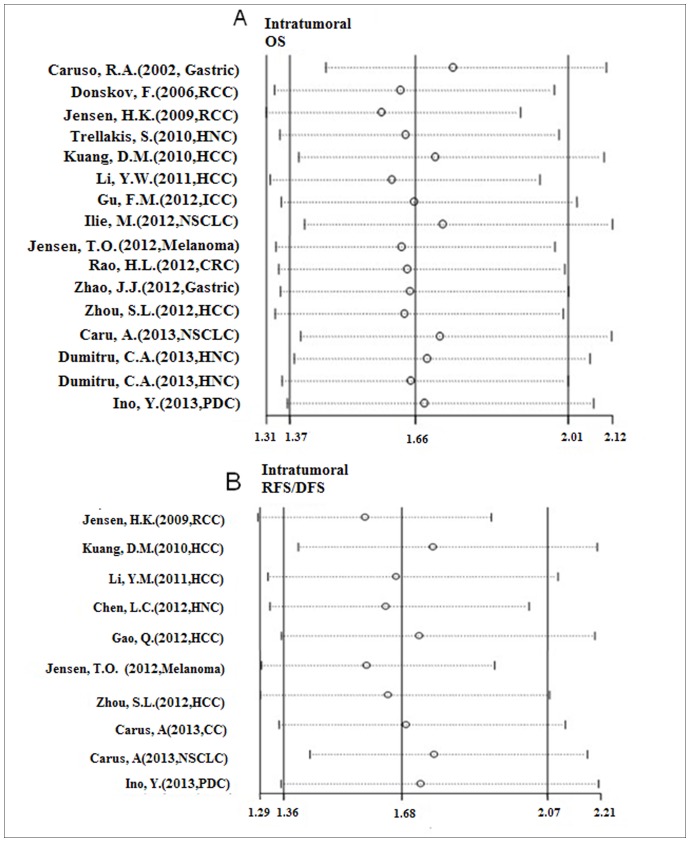
Sensitivity analysis of subgroups. (A) Sensitivity analysis of studies evaluated intratumoral neutrophils expression on OS. (B) Sensitivity analysis of studies evaluated intratumoral neutrophils expression on RFS/DFS.

Sensitivity analysis investigates the influence of a single study on the overall meta-analysis estimate, which computes the pooled HRs by omitting one study in each turn. The results of sensitivity analysis show whether the studies are convincing and stable. In our analysis, it demonstrated that all data assessing the prognostic role of intratumoral neutrophil levels in all cancer patients were stable with OS as the endpoint (Fig. 4A), also RFS/DFS as the endpoint (Fig. 4B).

## Discussion

To our knowledge, this is the first meta-analysis to analyze high levels of intratumoral neutrophils as an independent prognostic factor for short RFS/DFS, CSS, and OS in patients with cancer. Heterogeneity in patient outcomes to surgery or medical intervention has been a challenge for many years. This meta-analysis emphasizes a significant component of that heterogeneity came from differences at the level of neutrophil infiltration within the tumor microenvironment. The fact that different histological types of cancers all share in common the infiltration of neutrophils may point at TAN as a new prognostic factor in cancer that should be further assessed.

The implication of the meta-analysis is that the risk of recurrence or death is increased by at least 66% if elevated levels of intratumoral neutrophils are detected. Thus, patients with intratumoral neutrophils should have a closer follow-up. Intratumoral neutrophils may also serve as a new stratification factor in randomized trials. Moreover, the incorporation of intratumoral neutrophils into current prognostic models based on established and validated clinicopathological features remains a priority.

Inflammatory responses play an important role at different stages of tumor development, invasiveness, and metastasis. However, the role of neutrophils in the tumor microenvironment has been considered negligible until recently [4], in contrast to the well-characterized TAM [30]. Nevertheless, within recent years new data have emphasized an important role of TAN in cancer patients. These clinical observations are strongly supported by functional studies showing that cancer cells and/or other cells within the tumor microenvironment modulate neutrophils to infiltrate the tumor tissue and to acquire tumor-promoting activities, such as angiogenesis [32], migration, invasion, metastasis, mutagenesis or immunosuppression [33]. Neutrophils cross-talk with tumor cells through the production of cytokines and other molecules [34] and through the interaction with other cells in the tumor microenvironment [35]. Importantly, in well-established invasive human cancer the negative prognostic role of TAN has been surprisingly consistent and clear [36]. The present meta-analysis adds to this evidence and should prompt further research in this area.

In our analysis, we pooled 20 studies including 3946 patients and observed high densities of intratumoral neutrophils statistically associated with unfavorable OS. This was apparent for all cancers combined and for all subtypes of cancers except gastric carcinoma. The conflicting results in gastric carcinoma emphasize the importance of using modern IHC in the assessments of intratumoral neutrophils. Though the result in NSCLC was borderline value with 95%CI of 1.0–1.35, many meta-analyses would consider this statistically significant [37] [38]. More research is needed to validate results as the number of studies in each type of cancer was small. In addition, high levels of intratumoral neutrophils were significantly associated with short RFS/DFS and CSS especially in HCC. However, the pooled HRs of peritumoral and stromal neutrophils were not statistically significant. These results may suggest that neutrophils infiltrated in the tumor nests were the most accurate predictor of poor outcome in human cancer. However, the results obtained in the peritumoral and stromal compartments should be interpreted cautiously as the number of studies assessing neutrophils in various tumor compartment localizations was low (n = 4) and HRs for the peritumoral neutrophil had approximately the same level (HR range 1.66–1.8) as for the intratumoral neutrophil assessments. This may reflect the importance of neutrophil activity in the tumor nests and the migrating tumor border where the reciprocal communications between tumor cells and infiltrating neutrophils may be anticipated to be high and this should be further studied. However, systematic assessments of neutrophils in the different compartments, i.e, intratumoral, peritumoral and stromal, and the correlation of these cell subsets with other clinicopathological features and clinical outcome are cumbersome and technically challenging. It has been suggested that a simple estimate of the overall number of neutrophils relative to the overall number of CD8^+^ lymphocytes in the global tumor area is easier and faster and clinically translatable in terms of prognostic significance [29].

We assessed the impact of the primary antibody. Data indicated that CD66b positive TAN might have better prognostic value than CD15. However, in the subgroup which had OS as the endpoint, data should be interpreted cautiously as the number of studies assessing intratumoral neutrophils with the CD15 antibody was small (n = 2). Leukocytes express many cell surface markers, but CD66b is restricted to activated neutrophils [39] [40]. While CD15, also called sialyl Lewis x (sLex), is expressed on the surface of leukocytes [41], mainly in neutrophils, eosnophils, and part of monocytes, and do not reflect the activation status of neutrophils. In addition, CD15 has been demonstrated to be expressed occasionally on tumor cells [42], [43]. No study has assessed both antibodies in parallel.

The assessment methods of TAN were varied in the present study. Semiquantitative methods are easy and fast but subjective and inter-observer variations may exist. Stereology assessments are objective and reproducible but laborious, cumbersome and require specialized training. Digital image analysis (DIA) is an emerging, high-throughput method for automated quantitative assessments of immunostained sections but may still be sensitive to variation by tissue processing, IHC protocols, nonspecific staining, and definition of region-of-interest. However, for clinical relevance and applicability of TAN assessments more efficient methods are warranted. Recently, a comparative study demonstrated DIA protocols to provide fast, robust, and potentially clinically applicable results with prognostic information comparable to the considerably cumbersome stereology method [44].

Several sources of heterogeneity should be considered in the present study. Statistical heterogeneity may due to the differences in the types of carcinomas, the method and evaluation of staining, primary antibody, neutrophil localization, assessment method, cut-off criteria, cut-off value, the variables used for matching and adjustment. Though all mentioned potential biases, the data were stable and convincing, as demonstrated by sensitivity analysis and the trim-and-fill analysis.

We did not estimate the pooled HRs in all adjusted data as the HRs of some negative results were not reported in stepwise Cox regression analyses because they were not in the equation. Cut-off values were different in each study. 14 studies used the median or mean levels as cut-off values, and only 1 study used fixed cut-off value. It emphasizes the importance of confirming a “standardized” cut-off value for future studies. Furthermore, different primary antibodies and methods for evaluating staining might result in different results. Therefore, to improve the quality of researches, future standardized protocols are needed.

Our study has limitations. First, all eligibly literatures in this meta-analysis were published in English. This may under represent negative studies in the meta-analysis [45]. Second, several elements might affect the pooled HRs, such as the differences in the country of population, characteristics of patients, follow-up time, cut-off criteria, cut-off value and the variables used for matching and adjustment. Third, there were some minor differences between the exact HR and the extrapolated data, according to Tierney’s method [24].

## Conclusion

In summary, the meta-analysis demonstrated high levels of intratumoral neutrophils were significantly associated with unfavorable survival and recurrence in human cancer, especially in HCC and ICC, HNC, NSCLC and RCC. Moreover, the CD66b primary antibody might be a better choice for evaluating the prognostic role of TAN rather than CD15 since the CD66b-positive antigen is assigned to activated neutrophils. Further research to understand the mechanism and functionality of TAN in cancer are encouraged.

## Supporting Information

Checklist S1 **PRISMA Checklist.**
(DOC)
